# Reconciling operational realities with high-flexibility green hydrogen: A case study of Australia’s national hydrogen plan

**DOI:** 10.1016/j.isci.2026.117549

**Published:** 2026-09-25

**Authors:** Navin Bhardwaj, Kinley Dorji, Dezso Sera, Tara Hosseini, Nargessadat Emami, Thomas Moore

**Affiliations:** 1 Queensland University of Technology, School of Mechanical Medical and Process Engineering, Brisbane, QLD 4000, Australia; 2 Queensland University of Technology, School of Electrical Engineering & Robotics, Brisbane, QLD 4000, Australia; 3 Energy Transition Centre, Brisbane, QLD 4000, Australia; 4 CSIRO Energy, Private Bag 10, Clayton South, VIC 3169, Australia; 5 CSIRO Environment, Black Mountain Science & Innovation Park, Acton, ACT 2601, Australia

**Keywords:** green hydrogen, flexible loads, National Electricity Market, NEM, Integrated System Plan, ISP, Renewable Energy Zones, REZ, variable renewable energy curtailment, VRE, electrolyser degradation, gas crossover, specific energy consumption, SEC, flexibility paradox

## Abstract

Global energy transition roadmaps increasingly rely on green hydrogen as a flexible load to mitigate renewable curtailment and defer transmission investment, yet a gap remains between grid planners’ least-cost optimization and the engineering limits of large-scale electrolysis. This perspective examines the hydrogen-as-a-flexible-load strategy, focusing on technology-specific minimum stable loads and deep-flexibility risks in grid models. Australia is a pivotal case study: the draft 2026 Integrated System Plan (ISP) shifts to weekly production balancing to halve 2050 curtailment to 10%, yet relies on a technical floor unproven at scale. We identify a flexibility paradox in which electrolyzers are forced into high-frequency grid stabilization without accounting for efficiency penalties and degradation from sub-nominal cycling. Contrasting ISP targets with operational data, manufacturer specifications and project reports, we distil six variables for planning models and identify the research and policy pathways needed to keep hydrogen’s grid role viable and sustainable.

## Graphical abstract

**Figure:**
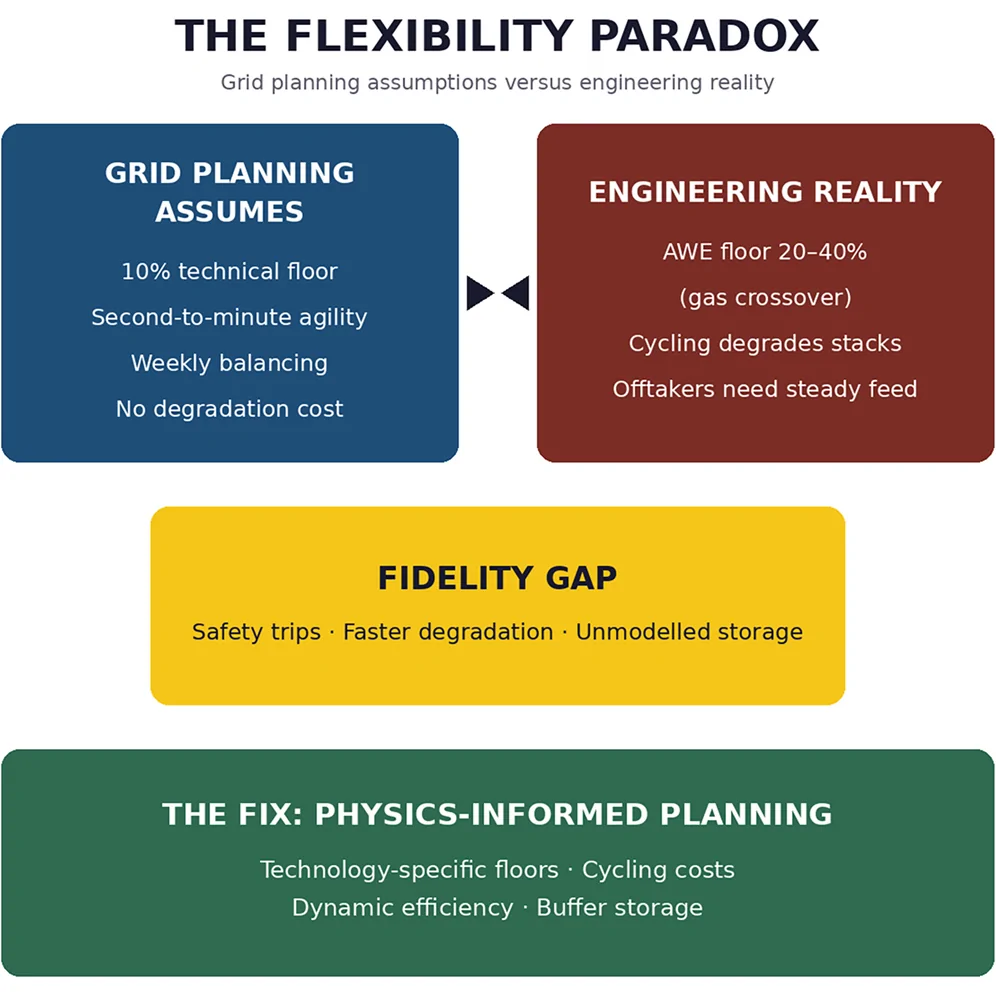

## Teaser

Applied sciences; Energy Resources

## Introduction

The global transition to net-zero emissions has made green hydrogen a cornerstone of macro-scale energy planning. As high-renewables jurisdictions retire synchronous thermal generation, focus has shifted to sector coupling, using industrial loads^1^^,^^2^^,^^3^ to balance wind and solar variability; central to this strategy is the electrolyzer as a flexible load,^4^^,^^5^ theoretically able to ramp in real time to absorb surplus generation and provide essential grid services.

Internationally, hydrogen infrastructure planning is converging on “hubs” and “backbones”: China’s resource-push corridors such as the Sinopec West-to-East pipeline,^6^^,^^7^ India’s planned National Green Hydrogen Grid,^8^ and the European Hydrogen Backbone, a planned 23,000 km network of largely repurposed gas pipelines.^9^^,^^10^ In each case, high-capacity transmission and its inherent line-pack storage (the gas volume held by pressurizing a pipeline above its delivery pressure) act as the critical decoupler that harmonizes volatile renewable generation with the rigid, steady-state requirements of industrial thermochemical processes.^11^

This perspective focuses on Australia, whose transparent, iteratively consulted roadmaps invite external technical critique. The Australian model has matured into a hub-and-spoke architecture centered on industrial ecosystems such as Gladstone, the Hunter Valle,y and the Eyre Peninsula^12^^,^^13^ (Figure 1); unlike the EU’s interconnected continental mesh,^9^ it links Renewable Energy Zones (REZs) to deep-water ports and green-metal processing zones through common use infrastructure.^16^

**Figure 1:**
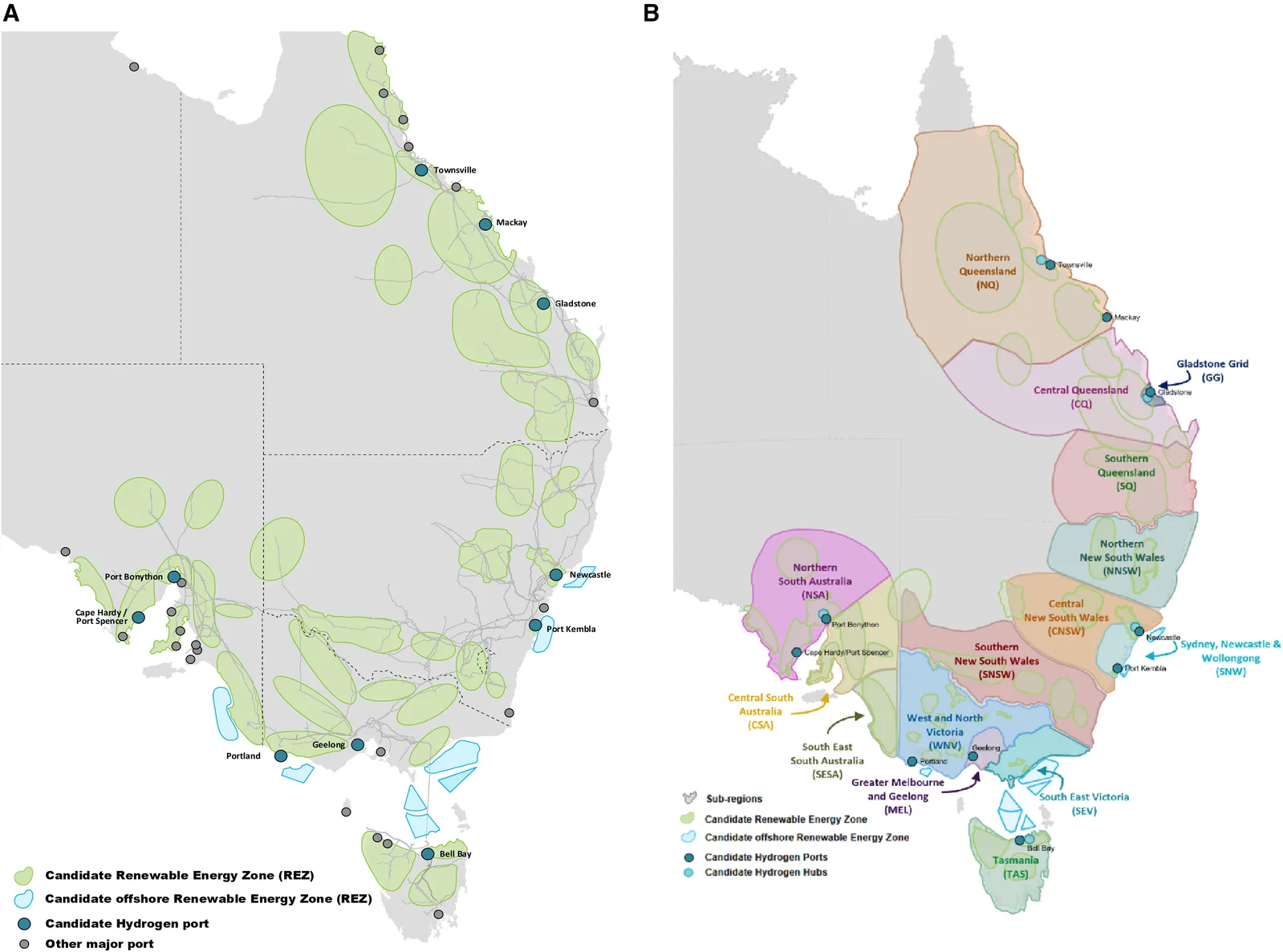
Evolution of the Australian hydrogen hub architecture between the 2024 and Draft 2026 Integrated System Plans (A) The ISP 2024 plan treats key ports as centers of hydrogen production and export or consumption, located in the proximity of renewable energy zones.^14^ (B) The Draft ISP 2026 plan evolves to identify key hydrogen hubs as centers of industrial consumption for hydrogen produced at electrolyzer stations co-located with the electricity generators at renewable energy zones, a strategic shift from a port-centric export model to a REZ-co-located hydrogen-hub model. This figure is adapted from AEMO.^14^^,^^15^

REZs are high-quality resource areas where clusters of large-scale renewable projects centralize generation; the 2026 Integrated System Plan (ISP) methodology scores candidate zones on resource quality, network proximity, social license and land use, and system strength to identify the optimal development path that maximizes net market benefit.^17^

The *ISP*, published biannually by Australian Energy Market Operator (AEMO), is the authoritative whole-of-energy-system blueprint for cost-effective investment over a 20-year horizon; notably, it lacks an explicit national hydrogen transmission backbone,^18^^,^^19^ leaving transport and storage with individual developers. Australia’s strategy has evolved from the “virtual battery” framing of 2018,^20^^,^^21^ through the export-focused 2019 National Hydrogen Strategy,^12^ to the 2024 Strategy’s renewable-only production and $2/kg production tax incentive targeting green metals.^13^^,^^16^^,^^22^

Table 1 summarizes the key changes from ISP 2024 to Draft ISP 2026 relevant to green hydrogen.

**Table 1:** Key changes from ISP 2024 to ISP 2026

	2024 ISP – Base case	Draft 2026 ISP—base case	Comments
2050 electrolyzer capacity	15 GW	4.5 GW	significant capacity reduction to align with the proposed domestic downstream consumers for green hydrogen
2050 VRE curtailment	∼20%	∼10%	by using electrolyzers as a means of absorbing surplus energy, the 2026 ISP effectively halves the projected spilled renewable energy
Balancing window	monthly	weekly	the 2026 methodology allows for weekly production targets as they are much better aligned with the needs of the downstream consumer sectors that ISP2026 is targeting
Transmission saving	minimal (baseline build)	significant avoided costs (up to 20%)	flexible hydrogen producing loads co-located with the power generators in the REZs reduce the need for overbuilding transmission lines to manage peak solar/wind export from REZs
Hydrogen exports	key priority	no longer a priority; focus on domestic industrial consumers	shift from shipping green energy abroad to decarbonizing local hard-to-abate sectors like green steel and chemicals

The strategic maturity is most evident in AEMO’s planning evolution. The 2024 ISP^23^ treated green hydrogen as a perfectly flexible, price-chasing commodity balanced monthly; the 2026 ISP^18^ adopts weekly targets, minimum utilization factors (near 70% in the 2030s, easing to 35% by 2050) and REZ co-location,^14^^,^^15^ cutting required grid investment by roughly 20% and positioning hydrogen as a provider of fast Frequency Control Ancillary Services (FCAS), with CSIRO GenCost^24^ projecting Proton Exchange Membrane (PEM) electrolyzers dominant in later years.

Under the step change scenario, the Draft 2026 ISP formalizes the hydrogen hub architecture, co-locating production within REZs (Figures 1A versus 1B). Projected 2050 electrolyzer capacity has been revised down from 15 to 4.5 GW (Table 1)^19^^,^^25^; this 70% reduction reflects a pivot from high-volume export ambitions toward a conservative, domestic-centric industrial application.

ISP 2026 positions green hydrogen as a primary tool for REZ viability, utilizing electrolyzers to actively manage curtailment and replace multibillion-dollar investments in power transmission infrastructure with relatively lower-cost hydrogen transmission pipelines to the points of consumption. This halves projected 2050 curtailment (Table 1), from 20% in the 2024 ISP^23^ (section 4.3, “Six times today's utility-scale wind and solar”) to just 10% in ISP 2026^18^ (section 5.2, “120 GW of grid-scale wind and solar”).

A fundamental change in the 2026 modeling logic is the move from monthly to weekly production balancing^17^ (section 2.4.8, “Modelling hydrogen in the capacity outlook model”). The 2024 ISP allowed electrolyzers “perfect foresight” over a 30-day window^26^ (section 2.5.2, “Modelling hydrogen in capacity outlook models”), yielding idle-then-intense operating patterns that industrial stakeholders criticized as unfeasible; the 2026 update tightens the window to 7 days and introduces minimum utilization factors. This maturation sharpens a growing flexibility paradox: the grid requires deep, rapid load-shifting, yet industrial electrolyzers are governed by rigid chemical engineering constraints.

Whether thousands of megawatts of electrolyzer load can seamlessly drop to a 10% technical floor^15^ remains untested at industrial scale; the underlying turndown and degradation constraints^27^^,^^28^ are examined in the flexibility paradox: ISP assumptions versus operating experience.

The contribution of this perspective is deliberately specific. Electrolyzer flexibility, degradation, gas crossover, and hydrogen storage each have established literatures; what has been missing is their confrontation with a live national planning instrument. We benchmark the concrete operational assumptions of Australia’s Draft 2026 ISP, namely its technical floor, weekly balancing window, utilization trajectories and the absence of dedicated buffer-storage modeling, against manufacturer-specified operating envelopes and the published, as-engineered performance of funded projects, and we translate the mismatch (the flexibility paradox) into named variables that national capacity-expansion models should carry.

## Approach and evidence base

This Perspective employs a cross-disciplinary analytical framework to evaluate the alignment between power system planning and electrochemical engineering. We compare Australia’s 2024 and 2026 ISPs to quantify shifting operational assumptions (the transition to weekly production targets, the 10% technical floor, and minimum utilization trajectories) imposed on the National Electricity Market (NEM) electrolyzer fleet. These macro-scale projections are benchmarked against manufacturer-specified operating envelopes,^29^^,^^30^^,^^31^ published project knowledge-sharing reports,^32^^,^^33^ peer-reviewed operational analyses,^34^ and reported performance data from operating facilities.^35^^,^^36^ By integrating established literature on gas crossover kinetics, thermal fatigue, and stack degradation, we qualitatively assess the physical risks inherent in deep-flexibility dispatch. This synthesis provides the basis for an evidence-based roadmap of research and policy interventions. Box 1, placed before the reference list, defines key terms for interdisciplinary readers.

Box 1 **Glossary of key terms**

**Table:** 

Term	Plain-language meaning
Technical floor	the lowest instantaneous power draw at which an electrolyzer plant can operate stably and safely
Minimum utilization factor	a long-horizon constraint on average annual production; distinct from the technical floor, which is instantaneous
LEL (lower explosive limit)	≈4 vol % H2 in O2; safety systems typically trip at 25%–50% of the LEL (1.6–2 vol %)
BoP (balance of plant)	auxiliary systems (power electronics, pumps, gas drying, compression) whose loads do not scale down with stack load
FCAS	frequency control ancillary services: fast grid-security services in the NEM
Line-pack	gas stored by pressurizing a pipeline above its delivery pressure
Multi-stack sequencing	reaching low plant-level output by switching individual stacks off while others run at high load
Dunkelflaute	“dark doldrums”: prolonged periods of simultaneously low wind and solar output

## The flexibility paradox: ISP assumptions versus operating experience

The step change scenario in the 2026 ISP represents the central planning benchmark for the NEM, characterized by a rapid, consumer-led transition that aligns with a 1.5°C global warming limit. It assumes the accelerated retirement of coal-fired generation and a massive scaling of renewable energy zones (REZs), underpinned by the electrification of heavy industry and the emergence of a domestic green hydrogen economy.

However, this scenario introduces a series of aggressive operational assumptions that represent a fundamental departure from previous grid modeling. We identify three primary technical friction points (1) minimum stable loads, (2) production balancing windows, and (3) downstream hydrogen use synchronization, which necessitate reconciliation between power system optimization and material science.

### The 10% turndown assumption: Gas purity and diaphragm integrity

A central pillar of the AEMO latest grid modeling is the classification of electrolyzers as highly agile, price-responsive loads. According to the 2025–2026 inputs, assumptions and scenarios workbook,^15^ new utility-scale electrolyzer hubs are modeled with a “technical floor” of just 10% of their nameplate capacity. This assumption allows the ISP to simulate hydrogen production as a near-perfect buffer that can remain operational at minimal consumption during peak price events or renewable droughts.

This assumption highlights a widening “fidelity gap” between power system optimization and electrochemical reality, a concern echoed by the Energy Systems Integration Group (ESIG), whose recent assessment of hydrogen in power system models warns that existing roadmaps miss high-fidelity operational characteristics, such as sub-hourly response limits and thermal recovery times, required for secure grid operation.^37^

As the largest operating green hydrogen plant in the world, the Sinopec Xinjiang Kuqa project^35^ offers the most visible reported illustration of this fidelity gap. Commissioned in 2023, the facility deployed 52 alkaline electrolyzers (260 MW) intended to soak up a volatile 361 MW solar photovoltaics (PV) profile. Publicly available operational data are limited and drawn primarily from press reporting; the account that follows carries that caveat. According to these reports, planning models (supported by supplier claims) assumed a 10% operational floor, but gas crossover forced a safety-critical turndown limit of 30%–50%, with operation below it triggering automated shutdowns and contributing to a reported initial utilization near 20%, consistent with independent Wood Mackenzie analysis attributing the low utilization to electrolyzers unable to operate safely below roughly half-load.^36^ Sinopec is reported to have pivoted by early 2026 to a stabilized-hybrid strategy of inter-stack sequencing (staggering stack operation to maintain high individual loads) and grid-firming. Kuqa therefore does not prove the assumed flexibility unachievable; it suggests that achieving it requires buffer storage or grid support that planning models must cost explicitly. Nor is the tension unique to one site: at the comparably configured 150 MW Ningxia Baofeng alkaline-on-solar plant, analyst reporting at commissioning questioned the achievability of its production claims and noted likely reliance on grid electricity when solar output is low, the same intermittency tension in a different form.^38^

Only one other project in the documented set publishes an as-engineered operating floor: Australia’s Yuri alkaline plant, whose Australian Renewable Energy Agency (ARENA) knowledge-sharing report specifies a minimum load of 40% per stack and 20% at plant level, as detailed in the 10% turndown assumption: gas purity and diaphragm integrity^32^ Kuqa and Yuri therefore anchor Table 2.

**Table 2:** Reported operating envelopes and project experience relevant to the ISP’s electrolyzer flexibility assumptions

Project (country; commissioned)	Technology, scale, and power source	Key reported or published finding	Evidence
Sinopec Kuqa (China; 2023)	260 MW alkaline (52 × 1,000 Nm3/h units) on 361 MW solar PV; grid-firmed after 2026 pivot	reported estimate: effective technical floor 30%–50%; initial utilization ≈20% (public project-level operational data unavailable); Pivot to stabilized-hybrid operation with inter-stack sequencing and grid-firming	industry reporting corroborated by analyst assessment^35^^,^^36^
Yuri (Australia; 2024)	10 MW pressurized alkaline (Peric) on solar PV + 8 MW battery, grid-connected	as-engineered technical floor: 40% per stack, 20% plant-level (the latter reached by shutting a stack down); 23–31 min warm-up; battery sized to buffer cloud events	public project report (ARENA knowledge-sharing)^32^
Envision Chifeng (China; July 2025)	500 MW electrolysis (Envision proprietary); 100% off-grid wind + solar	technical floor not published; world’s largest off-grid hydrogen-to-ammonia system (320,000 t/y); AI-coordinated microgrid and dynamic ammonia synthesis absorb wholly variable renewable supply; detailed operating envelopes not yet published	company announcement and industry reporting^39^^,^^40^
CEEC Songyuan (China; December 2025)	320 MW, phase 1; wind-powered	technical floor not published; dual ammonia/methanol offtake provides downstream absorption flexibility; 45,000 t H2/y planned at full build	industry reporting^41^
Jilin Electric Da’an (China; July 2025)	approximately 230 MW hybrid alkaline + PEM on 800 MW wind + solar via DC microgrid, with solid-state hydrogen storage	technical floor not published; flexibility engineered through the PEM/alkaline split and storage buffering; 32,000 t H2/y to ammonia; EU RFNBO-certified	industry reporting^42^
Ningxia Baofeng (China; 2021)	150 MW alkaline on 200 MW solar PV	technical floor not published; analyst reporting questioned production claims and noted likely reliance on grid electricity when solar output is low	industry and analyst reporting^38^
REFHYNE, Shell Rhineland (Germany; 2021)	10 MW PEM (ITM Power); grid-powered at refinery	technical floor not published; operated in highly responsive mode selling primary control reserve	public project report^43^
Fukushima FH2R (Japan; 2020)	10 MW single-stack alkaline (Asahi Kasei) on 20 MW solar PV with grid support	technical floor not published; purpose-engineered dynamic alkaline operation on fluctuating renewables, working a single large stack hard	manufacturer data and public project reporting (NEDO)^44^
Energiepark Mainz (Germany; 2015)	6 MW peak PEM (Siemens Silyzer 200; 3 units, each 1.3 MW continuous/2.1 MW time-limited peak); wind farm plus market-purchased grid power	technical floor not published; highly dynamic operation demonstrated (10% per second ramp, millisecond response; control-reserve prequalified), but peak stack loading is time-limited vs. the continuous rating, and offtake/storage logistics shape the operating profile	peer-reviewed operational evidence^34^
H2FUTURE, voestalpine (Austria; 2019)	6 MW PEM (Siemens); grid-powered at steelworks	technical floor not published; passed the transmission system operator’s qualification tests to sell the full range of grid frequency-support and backup reserve services, from second-scale to hour-scale response	public project report^45^
HYBRIT (Sweden; pilot 2021, storage 2022)	4.5 MW alkaline (Nel) + 100 m3 steel-lined rock cavern; fossil-free grid power	**t**echnical floor not published; storage decoupling of production from steelmaking demand; 26%–31% variable-cost savings demonstrated in practice (25%–40% projected at commercial scale); approximately 3,800 h at 94% availability	operator public project reporting^46^
HyP SA (Australia; 2021)	1.25 MW PEM (Siemens); grid-powered	no minimum operating load reported within the published operating envelope, 0%–100% ramp at 10% per second, under 1 min start-up	public project report (ARENA knowledge-sharing)^33^

This constraint is neither project- nor technology-specific, and international evidence brackets the ISP’s central assumption from both sides (Table 2). Peer-reviewed analysis of the 6 MW PEM Energiepark Mainz plant shows that even a highly dynamic system (10% per second ramps, millisecond response, prequalified for control-reserve markets) operates within system-level envelopes rather than stack limits alone: peak stack loading is time-limited relative to the continuous rating, and hydrogen offtake (gas-grid injection minimum flows, storage, and trailer logistics) shapes the operating profile.^34^ Its grid-service capability is echoed by the grid-reserve-prequalified PEM demonstrators REFHYNE and H2FUTURE^43^^,^^45^; Fukushima FH2R shows alkaline systems can be purpose-engineered for fluctuating input^44^; and Sweden’s HYBRIT pilot demonstrates the storage-decoupling solution this Perspective advocates, with variable-cost savings of 26%–31% demonstrated in practice and 25%–40% projected at commercial scale.^46^ Most recently, Envision’s 500 MW off-grid Chifeng plant (fully operational since July 2025, producing 320,000 tons per year of green ammonia from wholly variable wind and solar under AI-based microgrid control) indicates that whole-system co-design of generation, storage, and synthesis can deliver such flexibility at scale, although detailed electrolyzer operating data are not yet published.^39^^,^^40^ Deep, frequent flexibility is achievable, but only with technology-appropriate design and, in most cases, intermediate storage that the current ISP omits.

Notably, only two of the 12 projects in Table 2 publish an as-engineered technical floor; for the remainder, the operating envelope remains commercially confidential. Evidence entries in Table 2 distinguish four tiers (peer-reviewed operational evidence, public project reports, manufacturer data, and industry or analyst reporting), and the argument weights them accordingly.

Two distinct constraints must be separated in the ISP’s framework: the instantaneous minimum stable load (the 10% technical floor) and the long-horizon minimum utilization factor (near 70% in the 2030s, easing to 35% by 2050), an average-energy constraint reflecting offtake commitments and capital recovery. They act on different timescales and are not interchangeable: a fleet meeting a 70% annual utilization factor can still spend material time at the technical floor, precisely where the safety and degradation issues concentrate.

The minimum stable load of an electrolyzer is not a fixed number but is set by the physics of gas crossover relative to the lower explosive limit (LEL, approximately 4 vol % H_2_ in O_2_). In alkaline water electrolysis (AWE), the current leading technology for large-scale deployment, hydrogen-to-oxygen (HTO) crossover rises as load falls: less oxygen is produced while hydrogen permeation remains broadly load-independent, so the hydrogen-in-oxygen fraction increases, and industrial safety systems typically trip at 1.6–2 vol % (25%–50% of the LEL).^47^^,^^48^ For conventional industrial AWE, this places the reported minimum stable loads in the 20%–40% range.^11^^,^^48^ Peer-validated data at these deep turndown levels from industrial-scale plants remain scarce.^28^

By contrast, PEM electrolyzers carry no comparable electrochemical low-load floor at ambient pressure, owing to the membrane’s low gas permeability; their crossover constraint emerges at high differential pressure or with thinner high-performance membranes.^47^^,^^49^ Even so, commercial containerized PEM systems specify balance-of-plant (BoP)-driven minima that rise with plant scale: Nel quotes a 10% floor for its 1.25–2.5 MW units but a 20% floor for its 5–20 MW modular plants, below which the vendor’s engineering team must be consulted.^29^^,^^30^ More broadly, published datasheet envelopes are capability claims: the operating range a vendor will contractually warrant is negotiated project-by-project under confidential long-term service agreements and is scrutinized by the lenders’ technical advisors,^50^ so planning models calibrated to catalog specifications may overstate the flexibility that is contractually protected in practice. Anion Exchange Membrane (AEM) units share the alkaline crossover physics at the cell level (a single commercial module is floored at approximately 60% of nominal for safety) and achieve a wide system-level range only by staging many small modules.^31^ For PEM, the binding flexibility cost is instead the accelerated catalyst degradation and thermal fatigue induced by aggressive ramping and frequent start-stop cycling.^27^^,^^28^ This distinction matters for the ISP because its later-year fleet is modeled, per CSIRO GenCost,^24^ as predominantly PEM: there, the relevant unmodeled cost is degradation under cycling rather than a crossover floor.

These specification-level limits are confirmed at project level in Australia’s own funded portfolio. The ARENA knowledge-sharing report for the Yuri project (10 MW pressurized alkaline, Pilbara) states an as-engineered minimum load of 40% per stack and 20% at plant level, the latter reached by shutting a stack down, with warm-up times of 23–31 min and an 8 MW battery sized to buffer cloud events.^32^ Hydrogen Park South Australia’s 1.25 MW PEM unit, by contrast, ramps 0%–100% at 10% per second with no reported low-load floor,^33^ behavior consistent with PEM at small scale, low differential pressure, and modest BoP overhead. The ISP’s 10% system-level floor is therefore not reached by deep single-stack turndown but by multi-stack sequencing: switching individual stacks off within a hub while others remain at high load. Table 3 summarizes the technology-specific constraints.

**Table 3:** Technology-specific minimum stable loads and governing constraints

Technology	Reported single-stack minimum stable load	Governing constraint	Sources
Alkaline (AWE)	20%–40% for conventional industrial designs; ≈9%–12% achieved in a controlled research demonstration (not conventional commercial performance) with active pressure and lye-flow control at reduced safety margin	HTO crossover rises as load falls; safety trip at 1.6–2 vol % H2-in-O2 (25%–50% of LEL)	Buttler and Spliethoff^11^; Trinke et al.^47^; Oikonomidis et al.^48^; Li et al.^51^
PEM	no electrochemical floor at ambient pressure; commercial system minima are BoP-driven and rise with scale: 10% (1.25–2.5 MW) to 20% (5–20 MW)	crossover binding only at high differential pressure or with thin membranes; cycling-induced degradation dominates	Nel Hydrogen^29^; Nel Hydrogen^30^; Trinke et al.^47^; Omrani and Shabani^49^
AEM	≈60% for a single module (safety floor); ≈3%–105% at system level via modular staging	alkaline-type crossover physics at cell level; staging shifts burden to start–stop cycling	Enapter^31^
SOEC	reversible (−100% to +100%) but thermally constrained	must remain near 700°C–900°C operating temperature	Buttler et al.^11^

### The weekly balancing and the storage decoupler

The shift to weekly production targets forces the model to account for dark doldrums (Dunkelflaute), that is, prolonged periods of low wind and solar more accurately. However, this creates a secondary technical challenge: downstream process synchronization. Most primary off-takers for green hydrogen, such as Haber-Bosch plants for green ammonia or direct reduced iron (DRI) plants for green steel, are steady-state thermochemical processes. These plants cannot tolerate the deep load excursions, down to the 10% floor, implied by the sub-hourly curtailment-following dispatch that the 2026 ISP expects within each seven-day balancing window. The weekly window itself is an energy constraint and does not inherently mandate high-frequency cycling: three timescales must be distinguished, namely the weekly production target (an energy envelope), the sub-hourly price-responsive dispatch through which curtailment is absorbed, and second-scale FCAS response. The crossover and degradation risks examined here attach to the sub-hourly and second-scale layers; the storage requirement attaches to the weekly envelope.

To bridge this gap, the modeling must explicitly account for the CAPEX and infrastructure requirements of intermediate buffer storage. Without mitigation (whether buffer storage, production overcapacity, flexible synthesis operation, grid support, or hybrid hydrogen supply), the proposed flexibility poses a severe industrial challenge for steady-state offtakers. Two primary large-scale storage pathways exist, though both face significant inclusion gaps in the current ISP.

• The line-pack limitation: while utilizing the volumetric capacity of dedicated H2 pipelines is a standard buffer strategy (in connected networks such as the planned European Hydrogen Backbone, line-pack provides daily buffering at lower cost than geological storage^9^), the ISP 2026’s current hub and spoke architecture i.e., multiple REZ co-located hydrogen production sites servicing a proximal hydrogen hub without inter-hub connectivity, offers limited line-pack potential. Because the plan lacks a committed hydrogen transmission backbone to connect these isolated hubs, there is no large-volume pressurized network to absorb weekly supply-demand imbalances. This is clear from the assessment report for the required infrastructure that was completed in 2022.^52^ Indicatively, line-pack adds negligible marginal cost once a pipeline exists, whereas Australian salt-cavern storage is estimated at US $0.11–0.14 per kg of working capacity for best-case monthly cycling, rising to ≈ US $0.70 per kg for annual cycling^53^
• Geological storage and the weekly drought: in the absence of a pipeline backbone, geological storage, specifically salt caverns, depleted gas fields or lined rock caverns, becomes the leading large-scale option for surviving a seven-day renewable drought; above-ground compressed storage, chemical carriers such as ammonia, production overcapacity, and flexible offtake operation can contribute at smaller scales and shorter durations. While the published ISP documentation does not describe dedicated hydrogen buffer-storage modeling,^17^^,^^18^ active exploration of the Adavale Basin in Queensland for salt cavern suitability highlights that this is a critical, albeit unmodeled, infrastructure requirement.^53^^,^^54^

### Degradation penalties

Depending on plant configuration and control strategy, multi-stack sequencing buys plant-level flexibility at the cost of standby losses and repeated thermal cycling of the sequenced stacks. Each sequencing cycle substitutes one problem (low-load crossover) for another, frequent thermal cycling and pressure shocks that accelerate mechanical degradation of diaphragms and electrodes,^55^ a cost not yet priced into the ISP’s levelized cost of hydrogen (LCOH) projections. This degradation cost is not hypothetical. Unlike a lithium-ion battery designed for 5,000+ cycles, an industrial stack is engineered for steady-state longevity (80,000+ hours), and durability testing under dynamic operation shows measurable decay from cycling^56^^,^^57^^,^^58^: square-wave cycling at low catalyst loading produced a 142 mV overpotential rise over 31,500 cycles (≈4.5 μV per cycle), with losses increasing at higher cycling frequency,^56^ while long-duration testing frames a baseline decay near 28 μV per hour against field reports exceeding 100 μV per hour under dynamic operation^57^; each excursion to the technical floor may shave stack life through catalyst leaching in PEM systems or diaphragm stress in alkaline systems,^27^^,^^28^ a feedback loop absent from dispatch models. Degradation need not be represented solely as a fixed cost per cycle: state-of-health and equivalent-life-consumption formulations, analogous to those used for battery dispatch, offer alternative representations for planning models.^59^

### The efficiency penalty of grid ramping

Finally, we must address the efficiency-versus-flexibility trade-off. Electrolyser efficiency (kWh/kg H2) is not linear. Frequent ramping to follow the solar duck curve forces the plant to operate frequently in the transient phase where auxiliary loads (cooling, drying, and compression) consume a higher percentage of total power. Operational data from the Siemens PEM system at Energiepark Mainz show that system efficiency, including all auxiliary consumers, is comparatively flat across the mid-load range but falls steeply at low part-load, where fixed BoP consumption becomes a large share of input power^34^ (Figure 2); transient operation imposes an additional penalty that the steady-state curve does not capture. For the Mainz system this penalty is material: ramping down from the rated 4.5–0.45 MW reduces whole-system efficiency from approximately 63% to around 50% (Figure 2), and the penalty would be significantly higher if a lower technical floor were targeted. It is also worth pointing out that these systems are often operated to optimize for peak production with low degradation penalties rather than peak efficiency.

**Figure 2:**
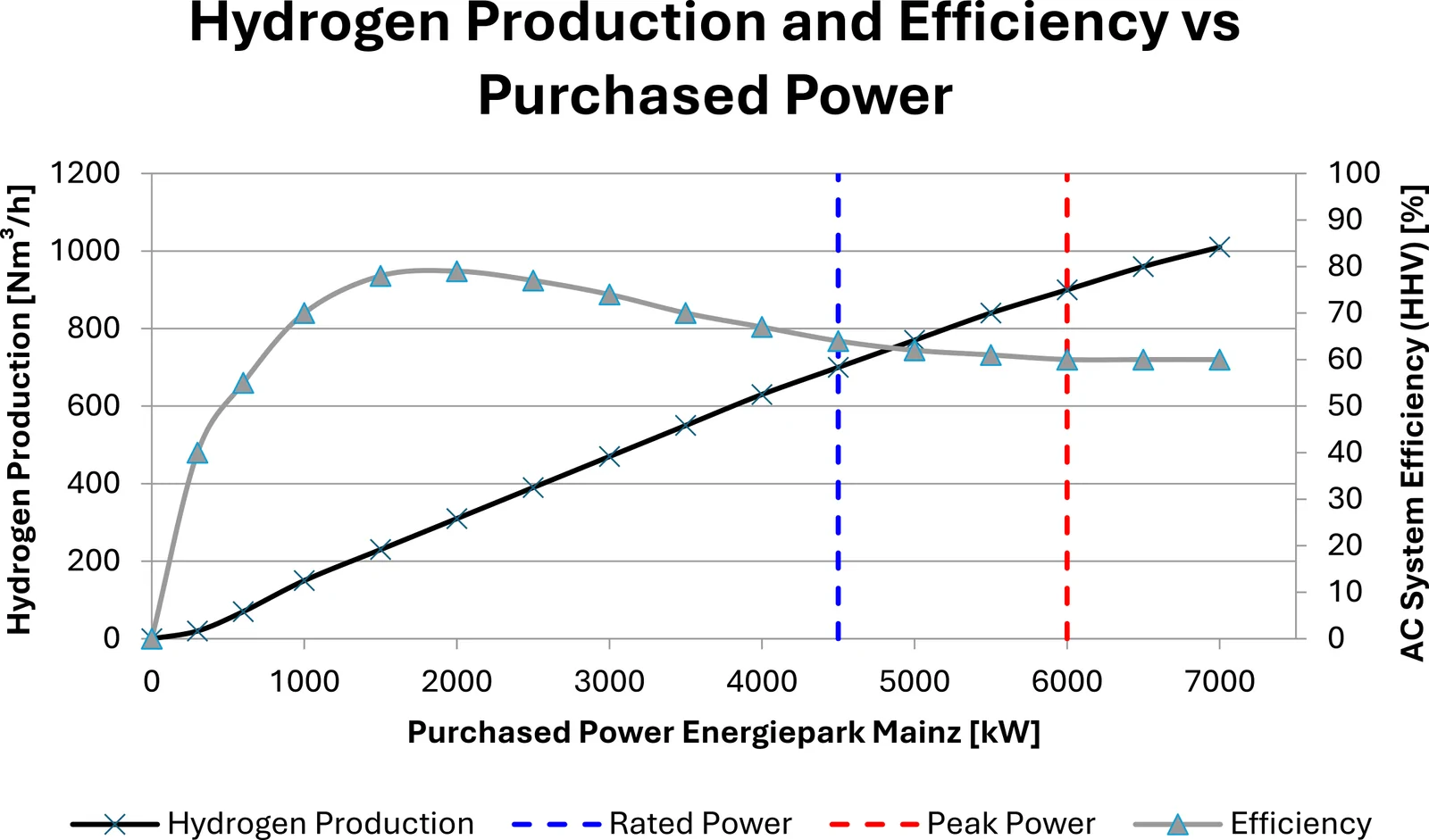
Efficiency of the Siemens PEM electrolysis system at Energiepark Mainz, including all auxiliary consumers (compression, cooling, water purification, control) The curve reports steady-state part-load performance; the steep decline is confined to low part-load, where fixed balance-of-plant loads dominate. This figure is adapted from Kopp et al.^34^

If the electrolyzer is constantly ramping to soak up curtailment, the specific energy consumption increases. This reduces the total hydrogen yield per unit of consumed energy, meaning the 2,050 curtailment absorption of 10–12 TWh projected by AEMO^18^ might actually produce less usable fuel than anticipated.

## Knowledge gaps

Current energy-system models treat electrolyzers as black-box assets with highly simplified flexibility, while materials research focuses on cell-level degradation at steady state; the missing link is how macro-grid requirements, such as the 168-h balancing window, translate into micro-scale degradation when a multi-stack plant is driven toward its 10% system-level floor.

Three gaps follow. Volumetric storage: if the electrolyzer drops to 10% load while a green ammonia plant requires steady feed, buffer storage must be sized for the worst-case weekly production deficit, a capacity absent from the ISP’s cost stack. Multi-stack control: BoP base loads (power electronics, cooling, gas drying) do not scale down with output, so running a 100 MW facility at 20 MW output is inefficient and risks thermal imbalance across the manifold. Degradation accounting: the cycling-life feedback identified in degradation penalties remains unpriced in national dispatch models. Table 4 summarizes these constraints against the corresponding ISP 2026 modeling assumptions.

**Table 4:** Comparison of grid modeling assumptions (AEMO 2026 Draft ISP) vs. industrial engineering constraints

Parameter	2026 ISP modeling assumption	Industrial/Engineering reality	Impact on grid reliability
Minimum stable load (turn-down)	10% (facility level)	technology- and scale-dependent: AWE 20%–40% per stack; PEM 10%–20% (BoP-driven, rising with scale); AEM ≈60% per module (Table 3)	unplanned trips if load is forced below the safe turndown floor; the grid loses flexibility the model assumes is available; forced stack shutdowns add thermal-cycling degradation
Safety thresholds	not explicitly modeled	gas purity (LEL) limits	forces hard shutdowns during high-curtailment events if loads drop too low, removing the flexible load exactly when the grid needs surplus energy absorbed
Production balancing	weekly (7-day window)	hourly/continuous (process dependent)	overestimates ability to stay idle during long renewable energy droughts without storage
Downstream synergy	fully decoupled (independent load)	process-coupled (ammonia/steel)	downstream reactors require steady feed; necessitates significant investment in H2 storage
System efficiency	static/optimized	dynamic/degrading	overstates delivered hydrogen per MWh absorbed; ignores parasitic load increases and cumulative stack degradation during frequent ramping

## Future outlook and concluding remarks

The shift toward weekly balancing in the 2026 ISP represents a significant step toward realistic grid planning, yet it also reveals the limitations of the state-of-the-art power systems modeling tools when applied to water electrolysis. As the energy sector pivots from pilot projects to gigawatt-scale deployment, the next decade of research must move beyond least-cost capacity modeling and toward physics-informed operational strategies.

Concretely, six variables should enter national planning frameworks, each grounded in the evidence assembled in the flexibility paradox: ISP assumptions versus operating experience section: (1) technology-specific minimum stable load, at both single-stack and plant level^29^^,^^30^^,^^31^^,^^32^^,^^47^^,^^48^; (2) start-stop and thermal-cycling penalties, expressed as cycle-count limits and a cost per cycle^31^; (3) a degradation term in the dispatch objective (whether formulated as a cost per cycle, a state-of-health trajectory or equivalent-life consumption)^55^^,^^59^; (4) load-dependent dynamic efficiency curves in place of static efficiency^34^; (5) buffer storage sizing coupled to the balancing window and dimensioned for the worst-case weekly production deficit^53^^,^^54^; and (6) downstream feed-stability constraints for process-coupled offtakers.

Degradation-aware dispatch is maturing rapidly,^55^^,^^59^ but an implementation gap remains before adoption in frameworks like the ISP, and whether grid-optimal flexibility is also economical for the asset owner remains open. That question is now live on the international policy agenda: the UK government’s 2026 call for evidence on electrolyzer flexibility acknowledges that “operating only during periods of surplus renewable generation is not commercially viable under existing business models.”^60^ Flexibility-ready stacks (robust diaphragms, ramp-tolerant catalysts) and digital-twin test beds simulating 20 years of weekly balancing must validate performance at low regret cost before gigawatt-scale deployment. Australia’s Draft 2026 ISP offers a global template for sector-coupled planning, yet its projected savings still rest on a degree of electrolyzer flexibility that the underlying electrochemistry has yet to demonstrate at scale; closing that gap through physics-informed dispatch and buffer storage will determine whether hydrogen can serve as a sustainable cornerstone of the energy transition.

## Acknowledgments

N.B. acknowledges the School of MMPE at Queensland University of Technology and CSIRO for providing a top-up scholarship in support of this research. The authors gratefully acknowledge Nel Hydrogen and Deepak Mistry (Pacific Energy, Nel representative in Australia) for confirming the minimum-load (technical floor) specifications of Nel electrolyzers and for clarifying that published datasheets provide capability guidance only, with warranty-backed performance guarantees negotiated separately under project-specific long-term service agreements. Any interpretation of this information is the authors’ own. This research did not receive any specific grant from funding agencies in the public, commercial, or not-for-profit sectors. Support was provided through the authors’ respective institutions.

## Author contributions

N.B., conceptualization, methodology, investigation, formal analysis, visualization, and writing – review and editing; D.S., supervision, conceptualization, methodology, investigation, validation, and writing – review and editing; T.H., conceptualization, methodology, and writing – review and editing; N.E., supervision, conceptualization, and writing – review and editing; T.M., supervision, conceptualization, methodology, investigation, validation, and writing – review and editing; K.D., methodology, investigation, resources, and writing – review and editing.

## Declaration of interests

The authors declare that they have no known competing financial interests or personal relationships that could have appeared to influence the work reported in this paper.

## Declaration of generative AI and AI-assisted technologies in the writing process

During the preparation of this work, the authors used Gemini (Google) to assist with language refinement and improvement of readability. After using this tool, the authors reviewed, edited, and verified all content and take full responsibility for the content of the published article.
